# Perioperative anesthesia management of high-molecular-weight kininogen deficiency: a case report and literature review

**DOI:** 10.3389/fmed.2025.1678906

**Published:** 2026-01-06

**Authors:** Tao Jiang, Guancheng Song, Yuchao Chen, Liang Zhang, Yizhi Xu

**Affiliations:** 1 Department of Anesthesiology, Chongqing Traditional Chinese Medicine Hospital, Chongqing, China; 2 Department of Hematology, Chongqing General Hospital, Chongqing University, Chongqing, China

**Keywords:** HMWK, hemostasis, APTT, blood coagulation factor, thrombosis

## Abstract

A prolonged activated partial thromboplastin time (APTT) is a common abnormal finding in preoperative coagulation screening. Failure to accurately identify its underlying etiology can lead to a misjudgment of bleeding risk, potentially resulting in surgical delays, unnecessary transfusion of blood products, or even invasive further investigations. These consequences, in turn, increase both healthcare costs and patient risk. The prolongation of APTT is attributable to a variety of pathophysiological states, including coagulation factor deficiencies and the presence of pathological inhibitors. Among these, High Molecular Weight Kininogen (HMWK) deficiency is an extremely rare autosomal recessive disorder, characteristically marked by a significantly prolonged APTT *in vitro*, yet without a corresponding clinical bleeding tendency. This study report provides a detailed account of the anesthesia management during the perioperative period in a patient with HMWK deficiency. The patient is a 27-year-old female who, due to a markedly prolonged APTT, underwent genetic testing that revealed a defect in the kininogen-1 gene (KNG1) and was diagnosed with HMWK deficiency. She was scheduled to undergo laparoscopic salpingostomy, ovarian lesion resection, and hysteroscopic endometrial lesion resection due to abnormal uterine bleeding, bilateral hydrosalpinx, and a left ovarian cyst. The patient did not receive prophylactic transfusion of blood products, such as fresh frozen plasma or coagulation factors, nor any pharmacological intervention during the perioperative period. No abnormal bleeding or thrombotic events were observed. This case report, incorporating a systematic literature review and based on the pathophysiological principle of intact hemostatic function in patients with HMWK deficiency, culminates in the formulation of a set of perioperative anesthetic management strategies for this patient population.

## 1 Background

In patients with hereditary coagulation disorders, surgical trauma and the influence of anesthetic factors during the perioperative period may lead to abnormal intraoperative bleeding. Therefore, thorough preoperative examinations and preparations are indispensable for such patients. As a crucial indicator for preoperative assessment of coagulation function, a prolonged APTT typically suggests that a patient may have a coagulation dysfunction, which could potentially increase the risk of bleeding during surgery (1). Hence, meticulous discrimination and analysis of such indicators should be conducted before surgery to facilitate the implementation of appropriate management measures. We have authored a case report on the anesthetic management of a patient with the rare condition of HMWK deficiency, aiming to enhance anesthesiologists’ ability to identify such patients, deepen their understanding, and refine their clinical management strategies.

## 2 Case report

A 27-year-old female patient, with a height of 157 cm and a weight of 65 kg, was scheduled for laparoscopic salpingostomy, ovarian lesion resection, and hysteroscopic endometrial lesion resection due to abnormal uterine bleeding, bilateral hydrosalpinx, and a left ovarian cyst. The patient has no family history of consanguinity within the last three generations. Notably, the patient underwent an uneventful laparoscopic appendectomy for acute appendicitis at an external institution 6 years prior, with no abnormal bleeding or thrombotic complications reported during the perioperative period. The patient had experienced prolonged menstrual cycles one year prior, with menstrual flow comparable to her usual period for the first 7 days, followed by persistent spotting lasting approximately 10 additional days before cessation. Examinations conducted at an external hospital revealed significantly prolonged APTT. The APTT correction test (both immediate and 2-h corrections) showed normal results, with factor XII (FXII) activity at 67% (Factor XII activity was measured using a coagulation assay kit on a Sysmex CN-6000 fully automated coagulation analyzer. The reference interval was 70–150%). The activities of factors II (FII), V (FV), VII (FVII), VIII (FVIII), IX (FIX), X (FX), XI (FXI), and von Willebrand factor (VWF, both antigen and activity) were all within normal ranges, while the lupus anticoagulant standardized ratio was 1.3. Genetic testing identified a homozygous KNG1 mutation (chromosomal location GRCh37chr3:186445089–186445090, variant information NM 00102416.3:c.628_629del (p. Asn210Phefs*15)), indicating HMWK deficiency. Despite the absence of abnormal coagulation symptoms in daily life, such as prolonged bleeding after injuries, gingival bleeding after brushing teeth, or abnormal skin ecchymosis, the patient’s preoperative coagulation profile showed markedly prolonged APTT (133.9 s, The APTT, using ellagic acid as the activator (Shanghai Sun Biotechnology), was determined on an automated coagulation analyzer following the standard operating procedure. The established reference range was 22–38 s.). Blood routine examination revealed normal platelet count and aggregation function, normal fibrinogen levels, and normal prothrombin time (PT), but mild anemia (hemoglobin: 108 g/L). Thromboelastogram (TEG) indicated abnormal coagulation function (R: 42.4 min, K: 11.8 min, angle: 18.3 deg., MA: 38.6 mm), suggesting a hypocoagulable state. Based on a hematology consultation that considered the patient’s heavy menstrual bleeding and the endometrial involvement of the surgery, an intraoperative bleeding risk was identified. Following consultation with the hematology department, it was also considered that the patient had an increased risk of intraoperative bleeding. Therefore, following a recommendation from the Department of Hematology to prepare fresh frozen plasma (FFP) preoperatively, the elective surgery was performed as scheduled the next day.

After entering the operating room, comprehensive monitoring was instituted, encompassing standard electrocardiography, non-invasive arterial blood pressure measurements, pulse oximetry, end-tidal carbon dioxide monitoring, and continuous core temperature surveillance. Furthermore, a radial artery catheter was inserted to facilitate real-time intraoperative assessment of arterial blood gasses. Baseline blood gas analysis prior to the procedure documented a hemoglobin concentration of 83 g/L. Anesthesia induction was achieved utilizing a regimen consisting of sufentanil (20 μg), propofol (150 mg), midazolam (2 mg), and cisatracurium (10 mg). Anesthetic maintenance involved a targeted depth of anesthesia, achieved through the combined administration of remifentanil, propofol, and sevoflurane. Patient temperature was maintained intraoperatively using a forced-air warming device. Following the commencement of the surgical procedure, continuous blood gas analysis was performed, and meticulous observation of the surgical site and operative field was carried out; no significant bleeding or anomalies were noted. Post-procedure blood gas analysis indicated a hemoglobin level of 81 g/L, corroborating minimal blood loss. Throughout the surgery, the patient’s vital signs remained stable. The total operative duration was 90 min. Intraoperative fluid management involved a total infused volume of 1,600 mL, with an observed urine output of 150 mL, and an estimated blood loss of approximately 50 mL. Following the conclusion of the surgery, the patient was transferred to the Post-Anesthesia Care Unit, where full recovery of consciousness was confirmed after 35 min of observation before transfer back to the ward. The lithotomy position required for the surgery was a notable concern, as prolonged immobilization of the patient’s limbs and flexion of the lower extremities would impede deep venous return and the potential exacerbation of this risk by prolonged immobility, preventative measures were implemented immediately upon the patient’s return to the ward. These included the initiation of intermittent pneumatic compression therapy and encouragement of active limb mobilization. Ambulation was commenced as early as 5 hours postoperatively. On the day of surgery, the patient experienced minimal vaginal bleeding, which was managed conservatively without specific intervention. A liquid diet was introduced on the first postoperative day, and prophylactic anticoagulation therapy was initiated 24 h postoperatively with a subcutaneous injection of low-molecular-weight heparin (LMWH) at 4000 IU. Postoperatively, no imaging studies or D-dimer assays were performed to rule out subclinical thrombosis. On postoperative day 3, the vaginal bleeding had almost completely resolved. The patient was discharged on the fourth postoperative day. Pre-discharge blood tests revealed an improved hemoglobin concentration of 88 g/L, and throughout the entire hospitalization, no clinically significant bleeding events or thrombotic complications were documented. A two-month post-discharge telephone follow-up revealed that the patient’s menstrual cycle and volume had returned to baseline, with no other bleeding or thrombotic events.

## 3 Discussion

HMWK deficiency is an extremely rare autosomal recessive genetic disorder (with an estimated global prevalence of approximately 1 case per 8 million individuals, and approximately 1 case per 7 million individuals in East Asia). It is caused by defects in the KGN1 gene. Most patients with this condition are asymptomatic, with occasional occurrences of epistaxis (nosebleeds) and ecchymosis (bruising), but without spontaneous bleeding or a propensity for thrombosis. The disorder is often discovered incidentally during preoperative screening, which reveals isolated prolonged APTT (2). Through a literature review, this case report summarizes the screening, diagnosis, and anesthetic management protocols for patients with HMWK deficiency during the perioperative period.

### 3.1 The physiological roles of CAS and HMWK

The contact activation system (CAS) of intrinsic coagulation is composed of HMWK, prekallikrein (PK), and factor FXII (3). This system can only be activated upon contact with negatively charged surfaces (such as glass, kaolin, sulfate esters, and collagen), hence the term “contact activation.” The contact activation of the coagulation cascade is initiated by factor FXII, which is converted into activated factor XII (FXIIa) upon binding to negatively charged artificial or biological surfaces. FXIIa activates PK through proteolytic action, forming plasma kallikrein (PKa). Subsequently, PKa accelerates the activation of FXII. This positive feedback loop amplifies the production of FXIIa and PKa (4–6). Furthermore, FXIIa activates factor FXI, ultimately initiating the intrinsic coagulation pathway, leading to thrombin generation, fibrin formation, and platelet activation. This activation cascade serves as the basis for the APTT assay (7).

**Figure 1:**
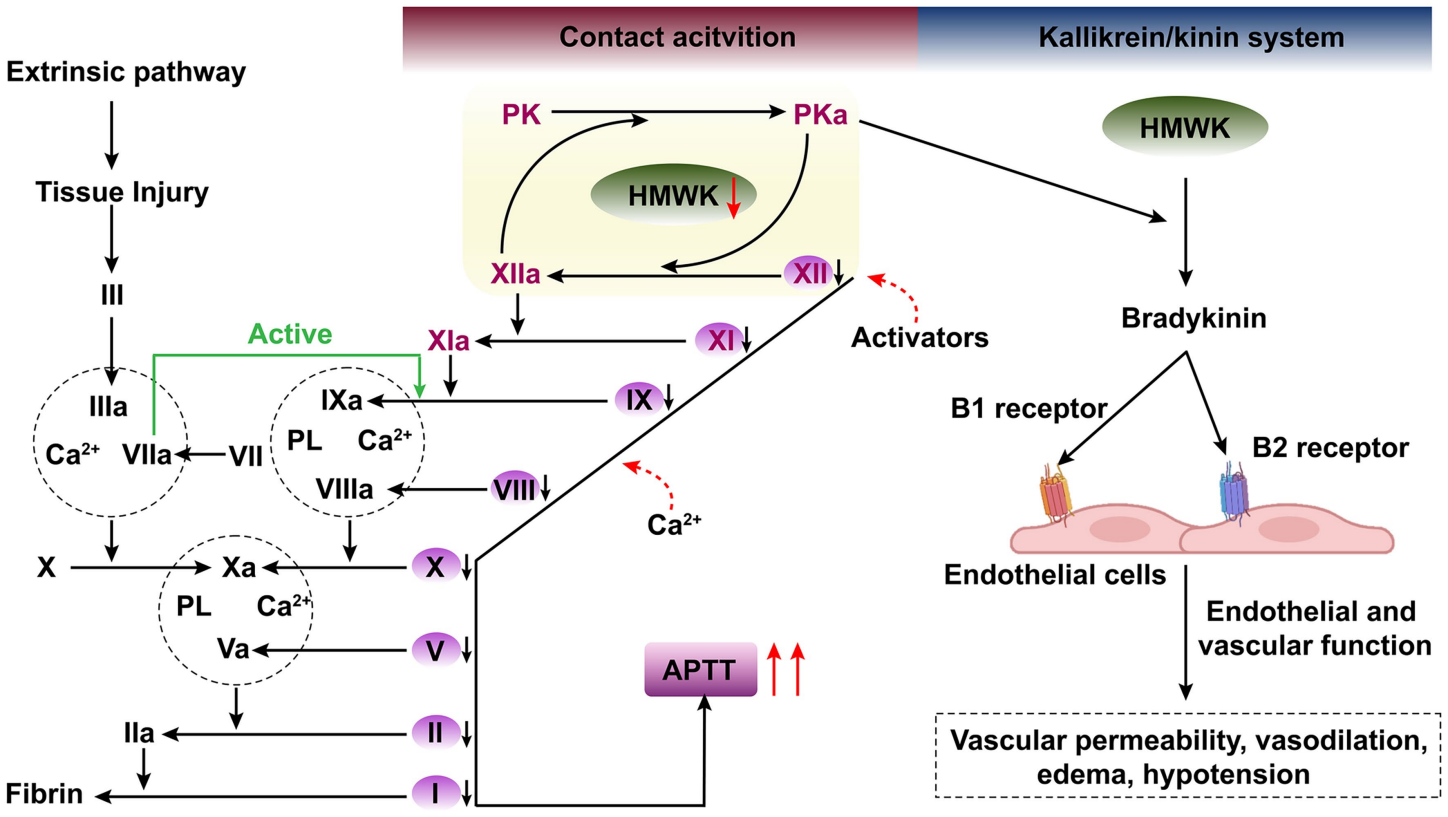
Schematic illustration of the activation of the intrinsic and extrinsic coagulation pathways, along with the depiction of the contact activation system (CAS) and the kallikrein-kinin system (KKS) within the vascular lumen and vessel wall. There exists a close interplay between CAS and KKS within the vascular lumen.

HMWK primarily participates in two physiological systems (Figure 1). Firstly, it forms the CAS together with three plasma proteins: coagulation factors XI and XII, and prekallikrein (PK), thereby participating in the activation of intrinsic coagulation (3). This system is initiated when contact factors (FXII, HMWK, PK) come into contact with negatively charged foreign surfaces. Within the CAS, HMWK serves as an essential carrier and cofactor, circulating in the plasma as non-covalent complexes with PK and factor XI (4, 5, 8). It localizes PK and factor XI adjacent to FXII on negatively charged surfaces, thereby facilitating blood coagulation and accelerating the activation of the intrinsic coagulation system (7). Secondly, HMWK, along with PK and factor XII, constitutes the kallikrein/kinin system (KKS) (7, 9, 10), which is implicated in inflammatory responses.

In the kallikrein-kinin system (KKS), prekallikrein (PK) and coagulation factor XII (FXII) are mutually converted into the protease plasma kallikrein (PKa) and activated coagulation factor XII (FXIIa), respectively (4, 11). PKa cleaves high-molecular-weight kininogen (HMWK) at two sites, releasing the peptide bradykinin (BK), which exerts its effects by binding to bradykinin B2 receptor (B2R) and bradykinin B1 receptor (B1R). BK plays significant physiological roles, including smooth muscle contraction, induction of hypotension, natriuresis and diuresis, and reduction of blood glucose levels. It also serves as a mediator of inflammation, causing and increasing vascular permeability and tension, releasing inflammatory mediators such as prostaglandins, stimulating nociceptors, and exhibiting both direct and indirect cardioprotective effects (12).

In addition, HMWK is involved in the fibrinolytic system, the renin-angiotensin system, the alternative complement pathway, and numerous other physiological functions (7).

### 3.2 Screening procedures for HMWK deficiency in patients with isolated prolonged APTT

As a member of the CAS, the deficiency of HMWK can lead to prolonged APTT, while also reducing the activity of PK, another component of CAS, thereby further prolonging APTT. This patient presented with isolated prolonged APTT at an external hospital, and the APTT correction test showed immediate and 2-h corrections. Further coagulation factor testing revealed no significant abnormalities in the activities of factors FII, FV, FVII-FXII, as well as in the antigen and activity of VWF. Therefore, the focus shifted to CAS, and genetic testing was conducted to identify a KNG1 mutation, providing an accurate diagnosis for the patient. The patient carried a homozygous frameshift mutation, c.628_629 (p. Asn210Phefs*15), in the coding region of the KNG1 gene, resulting from a non-triplet nucleotide deletion. This mutation led to the loss of normal protein function through nonsense-mediated mRNA decay (NMD) or premature termination of the encoded amino acid sequence. According to existing literature (2), all reported variants causing HMWK deficiency to date are truncating variants, NMD-inducing variants, or those severely affecting protein structure (splice site, nonsense, and frameshift mutations), rendering HMWK antigen (HMWK: Ag) undetectable. In this case, the patient’s mutation represents a newly discovered homozygous mutation site.

#### 3.2.1 Diagnostic criteria for HMWK deficiency

Given its rarity, there are currently no authoritative guidelines or expert consensus on HMWK deficiency. Since HMWK deficiency can lead to PK deficiency, the following diagnostic criteria, proposed by authors based on previous retrospective studies, can be used as a reference (2): (1) First, exclude other diseases that may cause isolated prolongation of APTT, such as hereditary or acquired factor deficiencies (when FXI levels are reduced, HMWK deficiency should be excluded), positive lupus anticoagulant, drug interference, and so forth; (2) PK activity or antigen levels below 5% or the presence of biallelic mutations in the KLKB1 gene are considered indicative of PK deficiency; (3) When PK activity or antigen levels range from 5 to 30%, PK or HMWK deficiency should be considered; (4) PK activity or antigen levels above 30% suggest the possibility of HMWK deficiency; (5) HMWK activity or antigen levels below 10% can serve as an auxiliary diagnostic criterion for HMWK deficiency, with final confirmation requiring the identification of mutations in both alleles of the KNG1 gene through sequencing.

### 3.3 Hemostatic and coagulation disorders in patients with HMWK deficiency

#### 3.3.1 Discussion on the bleeding risk in patients with HMWK deficiency

Physiological hemostasis initiates when tissue factor (TF) expressed by perivascular cells is exposed following vascular injury. Once TF is exposed to the bloodstream, it activates FVII, thereby initiating the extrinsic coagulation pathway. Following FVII activation, the common pathway factors FX and FV are sequentially activated, leading to the generation of a small amount of thrombin. FVIIa not only activates FX but also triggers the activation of factor FIX in the intrinsic pathway, thereby bridging the extrinsic and intrinsic coagulation pathways. This results in the massive production of thrombin, which is sufficient to convert fibrinogen into a fibrin meshwork capable of achieving hemostasis (13) (Figure 1). Notably, factor FXII, which is responsible for initiating the intrinsic coagulation pathway, along with HMWK and PK, are bypassed in physiological hemostasis, as they are not required for this process. Studies have demonstrated that when the prolonged APTT resulting from deficiencies in coagulation factors XII, PK, and HMWK, patients do not exhibit an increased bleeding risk and do not require blood product supplementation therapy (2, 14–16).

#### 3.3.2 Discussion on the thrombosis risk in patients with HMWK deficiency

**Figure 2:**
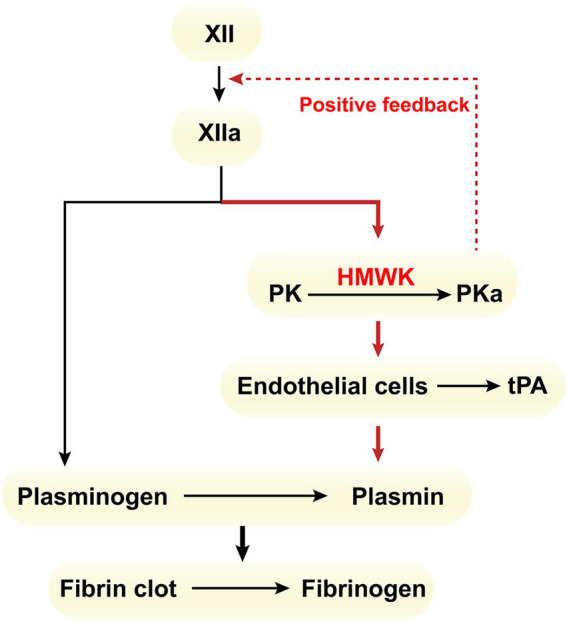
Intrinsic activation pathway within the fibrinolytic system.

As a cofactor, HMWK participates in the contact activation system on various negatively charged surfaces, which is implicated in thrombotic diseases and often secondary to contact activation triggered by infections caused by diverse pathogens (17). Clinically, there have been reported cases of HMWK deficiency accompanied by thrombosis (18–20). The primary mechanism by which HMWK contributes to pathological thrombosis is as follows: The intrinsic activation pathway of plasminogen (PLG) is predominantly mediated by factor FXIIa (21). In the direct pathway, FXIIa weakly activates PLG due to its serine protease domain homology with tissue-type plasminogen activator (t-PA) and urokinase-type plasminogen activator (u-PA). The indirect activation pathway, which is the predominant one, involves FXIIa, in the presence of HMWK, converting PK into its active form, PKa, which subsequently activates the fibrinolytic system (Figure 2). Severe HMWK deficiency results in low levels of PK (22) and reduced PK activity (3), thereby impairing PKa activation. This reduction in PKa activation leads to decreased secretion of t-PA by endothelial cells, diminished plasminogen activation, and impaired hydrolysis of fibrin clots, ultimately culminating in pathological thrombosis. A large prospective cohort study has also demonstrated a significant inverse correlation between HMWK levels and the incidence of venous thromboembolism (VTE) (23).

### 3.4 Perioperative management of patients with HMWK deficiency

HMWK deficiency is an exceedingly rare condition. Theoretically, patients with HMWK deficiency face a higher risk of thrombosis than bleeding. Several studies have reported cases of HMWK deficiency accompanied by thrombotic events, including lower extremity deep vein thrombosis and pulmonary embolism (18, 19). However, there is a paucity of data regarding the management of such patients during anesthesia and surgery. We conducted a search across databases such as MEDLINE (PubMed), EMBASE (Ovid), and Web of Science, and identified 16 case reports involving 21 patients with HMWK deficiency undergoing surgical procedures (2, 16, 24–37) (Table 1). Among these patients, 8 (8/21) had a history of bleeding prior to surgery. Data on the use of prophylactic measures, including medications or plasma transfusion, during the perioperative period were available for 5 patients (5/21), with another 5 patients (5/21) not receiving any prophylaxis; the prophylactic status of the remaining 10 patients was unclear. Postoperative bleeding occurred in two cases: a 34-year-old female experienced vaginal bleeding with hematoma and postoperative hemorrhage, and a 56-year-old female suffered from massive hemorrhage following hysterectomy for uterine fibroids. Only one thrombotic event was observed among the 21 patients, which was unrelated to surgery. This case involved a 64-year-old asymptomatic female who was found to have significantly prolonged APTT during routine preoperative evaluation and was subsequently diagnosed with HMWK deficiency. She underwent a successful cholecystectomy without any abnormal bleeding, and no thrombotic events occurred postoperatively. She died of pulmonary embolism at the age of 81. Although theoretical considerations suggest a prothrombotic risk in patients with High-Molecular-Weight Kininogen (HMWK) deficiency, no such thromboembolic events have been reported in the perioperative period in documented cases. Therefore, the prophylactic use of low-molecular-weight heparin (LMWH) in these patients is not advisable. Instead, a more appropriate strategy involves assessing the potential bleeding risk using Thromboelastography (TEG), and tailoring anticoagulation therapy based on the guidelines for the specific surgical procedure and the patient’s individual thrombotic history.

**Table 1:** Twenty-one patients with HWMK deficiency undergoing surgical procedures.

Case number	Author	Age	Sex	Bleeding	Prophylactic hemostatic response	Thrombotic events
1	Colman Williams	64	Female	N	N	Y
2	Anke Adenaeuer	87	Male	Y	Y	N
3	Stormorken	30	Female	Y	Y	N
4	Ahmad inejad	27	Female	Y	Y	N
5		31	Female	Y	N	N
6	Echenagucia	53	Female	Y	N/A	N
7		31	Female	Y	N/A	N
8		62	Female	Y	N/A	N/A
9		52	Female	N	N/A	N
10		55	Male	N	N/A	N
11	Hayashi Fujiwara	56	Female	N	N	N
12		56	Female	Y	N	N
13	Komiyama	34	Female	N	N/A	N
14	Lacombe Flaujeac	50	Female	N	Y	N/A
15	Lefrére	23	Female	N	N	N
16	Vincente	36	Male	N	N/A	N
17	Pancione	22	Male	N	N/A	N/A
18	Nakamura Tachibana	31	Female	N	N/A	N/A
19	Davidson	66	Male	N	Y	N
20	Xiaoying Lv	54	Female	N	N/A	N
21	Jennifer S Woo	71	Female	N	N	N

N, no; Y, yes; N/A, not available.

The patient we reported is a young female with HMWK deficiency and no prior history of bleeding or thrombosis. The surgical procedure involved disrupting the endometrium (which contains both pro-coagulant and fibrinolytic substances). The patient underwent hysteroscopic – laparoscopic surgery in the lithotomy position, with her limbs fixed for an extended period during the operation. The prolonged flexion of the lower limbs caused deep venous return obstruction, thus posing concurrent risks of bleeding and thrombosis. (1) Measures to address the bleeding risk: Although KNG-1 deficiency-induced HMWK deficiency typically does not cause bleeding, this particular case was caused by a novel homozygous variant of the KNG-1 gene, resulting in a significantly prolonged APTT of 133 s. The impact on her coagulation function remained uncertain. The relevant literature we reviewed still reported cases of perioperative bleeding. Moreover, the patient had prolonged menstrual cycles and increased menstrual blood loss before surgery, and the thromboelastogram results indicated a risk of hypocoagulable bleeding. Therefore, FFP and even suspended red blood cells were kept on standby before the elective surgery. (2) Measures to address the thrombotic risk: ① Tranexamic acid, which carries a risk of thrombosis, was not used during the operation (38). Instead, FFP was kept available as an alternative. ② Active thermal insulation measures were implemented during the operation to maintain the patient’s body temperature, as lower temperatures are associated with prolonged coagulation time (39). ③ Postoperative VTE prevention measures were taken: (a) In the early postoperative period, the patient was encouraged to turn over frequently and engage in bed-based activities. Elevating the feet helped promote venous return in the lower limbs. (b) The patient wore elastic compression stockings and had intermittent inflation-deflation of pneumatic compression bands wrapped around the legs to increase venous pressure in the lower extremities (40). (c) For pharmacological prophylaxis, low-molecular-weight heparin was administered after the operation. (d) A light and high-fiber diet was recommended to prevent constipation, and the patient was advised to maintain adequate fluid intake.

This patient was a young female with HMWK deficiency and no prior history of bleeding or thrombosis. A limitation of this case is the lack of perioperative dynamic monitoring of D-dimer and TEG, as only a single preoperative TEG was performed. The preoperative TEG showed a prolonged R time, often accompanied by abnormalities in parameters such as K time, Angle, and MA, which suggests a “hypocoagulable state” under conventional interpretation. However, this phenomenon is essentially an artifact caused by the high sensitivity of the *in vitro* testing system to the contact activation pathway. It differs significantly from the patient’s true *in vivo* hemostatic function, which is primarily driven by the tissue factor pathway, and thus does not represent a genuine bleeding risk (37). Consequently, prophylactic intervention specifically for HMWK deficiency is unwarranted. In clinical decision-making, the initial TEG result should be regarded as a baseline reference. Its core value lies in dynamic monitoring throughout the perioperative period to identify superimposed, genuine coagulopathies, such as fibrinogen or platelet dysfunction, rather than serving as a sole indication for initiating therapy (41). Furthermore, serial TEG monitoring can aid in the surveillance for postoperative subclinical thrombosis. Although whether HMWK deficiency increases postoperative thrombotic risk remains inconclusive, postoperative D-dimer and TEG testing should be performed. If any abnormalities are detected, they should be followed by further imaging investigations.

Given the unique nature of HMWK deficiency as a rare coagulopathy, it is challenging to establish safety standards for general anesthesia through large-sample studies based on the existing clinical evidence. Consequently, the cornerstone of perioperative management for patients with HMWK deficiency is the development and implementation of a preoperative, individualized risk assessment framework. This framework must comprehensively incorporate: a detailed medical history; genetic testing to elucidate the etiology and specific mutation profile; dynamic coagulation function monitoring, wherein TEG serves as a pivotal tool for quantifying potential bleeding risk; the application of validated bleeding and thrombosis risk scores; and a comprehensive evaluation of organ functional reserve. Furthermore, it is imperative to emphasize close collaboration within a multidisciplinary team, involving specialists from hematology, anesthesiology, surgery, and rehabilitation, to formulate meticulous intraoperative contingency plans and personalized postoperative rehabilitation strategies.

Firstly, it is crucial to enhance the awareness that HMWK deficiency leads to a prolonged APTT with a minimal bleeding risk, in order to prevent unnecessary surgical delays and unwarranted pro-hemostatic interventions. Secondly, the anticoagulation strategy for patients with HMWK deficiency must be precisely tailored based on procedure-specific guidelines and the patient’s individual thrombotic history, rather than on the diagnosis of HMWK deficiency itself. Furthermore, early postoperative mobilization is a routine, low-cost, and low-risk intervention; therefore, it is also recommended for this patient population, as it effectively balances the risks of bleeding and thrombosis, thereby facilitating rapid recovery. In this case, the patient initiated bedside mobilization on the day of surgery, which not only accelerated the recovery process but also laid a solid foundation for early discharge. This practice strongly demonstrates that incorporating early postoperative mobilization into the standardized management protocol for patients with HMWK deficiency has significant clinical implications for optimizing patient outcomes and enhancing the quality of care.

## 4 Conclusion

HMWK deficiency, as a rare coagulation disorder, typically does not directly precipitate abnormal bleeding or thromboembolic events during the perioperative period. Consequently, from a general prophylactic standpoint, the anesthetic plan typically does not require targeted modification, and routine pharmacological intervention or blood product transfusion is not required for patients with this condition. However, given the variability in individual coagulation status and the diversity of surgical procedures, a precise risk assessment is indispensable. TEG can generally assess the potential bleeding risk. Subsequently, an individualized and precise anticoagulation strategy should be formulated by integrating procedure-specific guidelines and considering the patient’s personal thrombotic history.

## Data Availability

The original contributions presented in the study are included in the article/supplementary material, further inquiries can be directed to the corresponding authors.
